# Review of the genus *Merulempista* Roesler, 1967 (Lepidoptera, Pyralidae) from China, with description of two new species

**DOI:** 10.3897/zookeys.77.938

**Published:** 2011-01-26

**Authors:** Yingdang Ren, Shurong Liu, Houhun Li

**Affiliations:** 1 College of Life Sciences, Nankai University, Tianjin 300071, P. R. China; 2 Institute of Plant Protection, Henan Academy of Agricultural Science, Zhengzhou 450002, P. R. China

**Keywords:** Lepidoptera, Pyralidae, Phycitinae, *Merulempista*, new species, China

## Abstract

The genus Merulempista Roesler, 1967 is reviewed for China. Of the four species treated in this paper, Merulempista rubriptera Li & Ren, **sp. n.** and Merulempista digitata Li & Ren, **sp. n.** are described as new; Merulempista cyclogramma (Hampson, 1896) is newly recorded for China, and its taxonomic position is briefly discussed. Photographs of the adults and genitalia are provided, along with a key to the known Chinese species.

## Introduction

Merulempista Roesler, 1967 is a small genus of ten described species and subspecies, distributed in the Palaearctic Region except Merulempista cyclogramma (Hampson, 1896) occurring in the Oriental Region and Merulempista oppositalis (Walker, 1863) ranging from Oriental to Australian regions (Roesler 1984; Asselbergs 1997; Leraut 2001, 2002). The genus is characterized by the male gnathos distally hooked, the sclerotized costa often produced to a distal process, and the female antrum deeply concave at middle and greatly extending backward posterolaterally.

Prior to this study, only Merulempista cingillella (Zeller, 1846) was known from China. The aim of the present paper is to review the Chinese Merulempista based on the specimens collected in Gansu, Guangxi, Hebei, Inner Mongolia, Ningxia, Tianjin and Xinjiang, describe two species new for science and record one species new for the Chinese fauna. All the specimens, including the types of the new species, are deposited in the Insect Collection, College of Life Sciences, Nankai University, Tianjin, P. R. China.

## Taxonomic accounts

### Merulempista

Roesler, 1967

Merulempista Roesler 1967: 274. Type species: Pempelia cingillella Zeller, 1846, by original designation.

#### Diagnosis.

**Figure 1.**
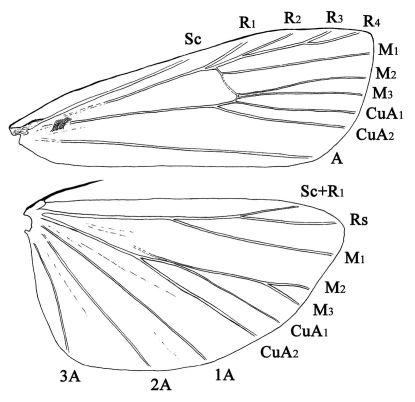
Wing venation. Merulempista digitata sp. n., paratype, slide No. LSR09042W.

Merulempista is similar to Meroptera Grote, 1882 by the forewing with M1 and M2 separated (Fig. 1), the third segment of the labial palpus rounded apically; the short robust gnathos hooked distally, the sclerotized costa of valva often with a distal process, and the narrow sacculus without spine. In Meroptera, M1 and M2 are shortly stalked on the forewing, the third segment of the labial palpus tapered distally, the gnathos is long and slender, the costa of valva lacks the distal process, and the sacculus has a spine (according to Roesler 1967).

#### Hostplants.

Tamaricaceae: Myricaria spp., Tamarix spp. (Huertas-Dionisio 2010).

#### Distribution.

**Figure 2.**
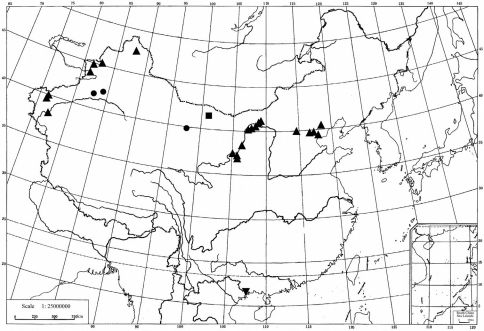
Distribution of Chinese Merulempista species. ■ Merulempista rubriptera, ● Merulempista digitata, ▲ Merulempista cingillella, ▼ Merulempista cyclogramma.

China (Fig. 2: Gansu, Guangxi, Hebei, Inner Mongolia, Ningxia, Tianjin, Xinjiang), Europe, North Africa and Australia.

#### Key to Merulempista species in China based on male genitalia.

**Table:** 

1	Costa sclerotized inconspicuously, without distal process; vinculum with a small papillate process anteromedially	Merulempista cyclogramma
–	Costa sclerotized conspicuously, with developed distal process; vinculum without papillate process anteromedially	2
2	Distal process of costa short triangular, without apical spine; phallus with three cornuti placed medially	Merulempista rubriptera
–	Distal process of costa somewhat fingerlike, bearing an apical spine; phallus with one cornutus placed medially, two placed distally	3
3	Distal process of costa almost straightly bending upward, forming a right angle with valva at outside	Merulempista cingillella
–	Distal process of costa obliquely bending upward, forming an acute angle with valva at outside	Merulempista digitata

### Merulempista rubriptera

Li & Ren sp. n.

urn:lsid:zoobank.org:act:46528BF5-9166-41A0-87E8-BDD379022DDF

Figs 3 7 11 

#### Type material.

Holotype ♂ – **China,** **Inner Mongolia Autonomous Region:** Erdaoqiao, Eji’naqi (41.56°N, 101.04°E), 927 m, 18.VII.2006, coll. Xinpu Wang and Xiangfeng Shi, genitalia slide no. LSR09083. Paratypes: 2 ♂♂, 2 ♀♀, same data as for holotype except dated 17–18.VII.2006.

#### Diagnosis.

This species is characterized by the rosy-colored forewing with a longitudinal greyish black stripe at base below costa. It can be distinguished from its congeners by the costa with a triangular distal process arising from 2/3 length of valva, the stout phallus with three thornlike cornuti located at middle in the male genitalia; and the antrum angularly protruding backward posterolaterally in the female genitalia.

#### Description.

**Figures 3–6.**
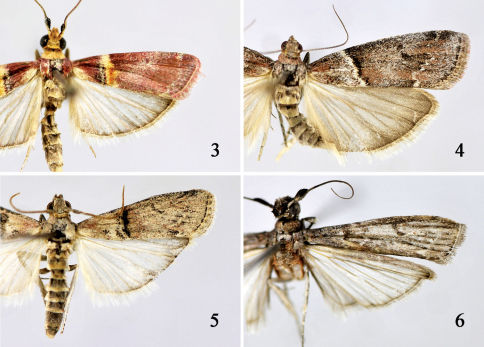
Adults of Merulempista species. **3** Merulempista rubriptera sp. n., paratype male **4** Merulempista digitata sp. n., paratype female **5** Merulempista cingillella male **6** Merulempista cyclogramma male.

Adult (Fig. 3). Wingspan 23.5–24.5 mm. Head khaki in male, pale yellowish brown in female. Antenna with scape reddish brown dorsally, yellow ventrally; flagellum yellowish brown ringed with brown; male sinus with shining deep greyish brown mixed with rosy scales, somewhat shelllike, pale yellow on ventral surface. Labial palpus pale yellowish white, densely covered with rosy scales ventrally, in female third segment tinged with black ventrodistally, far exceeding vertex; second segment shorter than diameter of eye, about 4 times length of third; third segment rather blunt apically. Patagium in male whitish yellowish tinged with pale yellowish brown, in female densely covered with greyish white-tipped rosy scales. Thorax and tegula in male rosy, in female greyish-brown tinged with rosy. Forewing rosy, with scattered greyish white and black scales in distal half, with a longitudinal greyish black stripe at base just below costa; posterior margin yellowish white at base; antemedian line yellowish white, straight, situated beyond basal 1/3, its posterior half tinged with black on inside, ocherous yellow on outside; postmedian and subterminal lines greyish white, slightly sinuate, nearly parallel; cilia rosy mixed with greyish brown, with a fine yellowish white basal line. Hindwing pale brown; cilia with basal 1/3 greyish brown, distal 2/3 greyish white. Legs rosy on outside, yellowish white on inside; tarsi brownish black, ringed with whitish yellow at apex of each segment, lined with short black spines on inside.

#### Male genitalia

**Figures 7–10.**
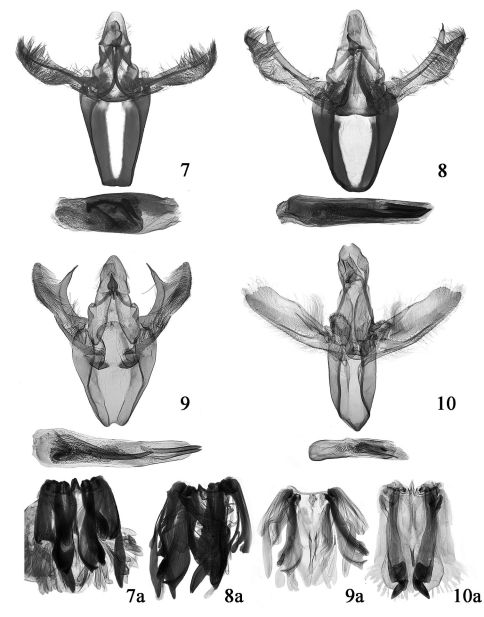
Male genitalia of Merulempista species. **7** Merulempista rubriptera sp. n., holotype, slide No. LSR09083 **8** Merulempista digitata sp. n., holotype, slide No. LSR09078 **9** Merulempista cingillella, slide No. DYL00263 **10** Merulempista cyclogramma, slide No. LJY10100 **7a–10a** 8th sternite and culcita.

(Fig. 7). Uncus somewhat trapezoid, with sparse setae laterally, blunt posteriorly. Gnathos slightly shorter than 1/2 length of uncus, heavily sclerotized, wide basally, tapering to hooked apex. Costa with distal process triangular, uprising obliquely outward from about 2/3 length of valva. Valva narrow basally, widened and arciform beyond middle ventrally, slightly narrowed toward bluntly rounded apex distally, densely setose; sacculus narrow, about 1/3 length of valva; clasper thumb-shaped. Vinculum large U-shaped, longer than valva, arciform anteriorly. Juxta slightly elliptical, concave deeply at middle on posterior margin. Phallus stout, about same length as valva; three thornlike cornuti located at middle of phallus, median one longest and curved basally. Eighth sternite and culcita shown in figure 7a.

#### Female genitalia

**Figures 11–14.**
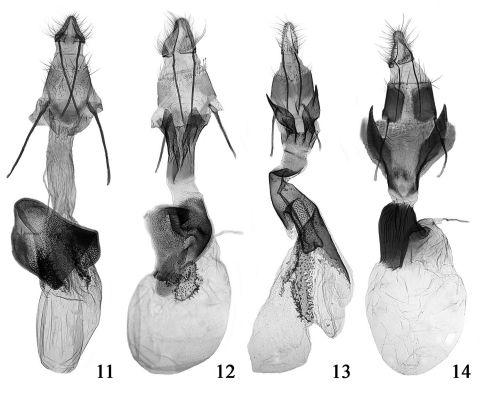
Female genitalia of Merulempista species. **11** Merulempista rubriptera sp. n., paratype, slide No. LSR09084 **12** Merulempista digitata sp. n., paratype, slide No. LSR09079 **13** Merulempista cingillella, slide No. DYL00279 **14** Merulempista cyclogramma, slide No. RYD04575.

(Fig. 11). Papillae anales triangular, narrowed to bluntly rounded posterior margin. Eighth abdominal segment longer than wide, anterior margin roundly protruding, posterior margin straight. Apophyses anteriores about as long as apophyses posteriores, slightly dilated at base. Antrum weakly sclerotized, deeply concave at middle on posterior margin, angularly protruding backward posterolaterally. Ductus bursae membranous, with longitudinal rumples. Corpus bursae membranous, elongate ovate; large, more or less rectangular accessory sac arising from left side of corpus bursae posteriorly, densely covered with granules, ductus seminalis from its apex; signa comprised of two clusters of short spines, placed posteriorly.

#### Distribution.

China (Inner Mongolia).

#### Etymology.

The specific epithet is derived from the Latin *ruber* (= red)and the suffix *–pteron* (= wing), in reference to the color of forewing.

### Merulempista digitata

Li & Ren sp. n.

urn:lsid:zoobank.org:act:DB61FC4B-ECD2-4F98-BE6D-D53BF581933D

Figs 1 4 8 12 

#### Type material.

Holotype ♂ – **China,** **Xinjiang Uygur Autonomous Region:** Xinyuan (43.19°N, 84.01°E), 1562 m, 7.VIII.2007, coll. Xinpu Wang, genitalia slide no. LSR09078. Paratypes: 22 ♂♂, 33 ♀♀, same data as for holotype except dated 6–7.VIII.2007; 1 ♂, Mohe, Gongliu (43.13°N, 82.45°E), 1500 m, 29.VII.1994, coll. Houhun Li and Hongyan Qin; 4 ♂♂, 5 ♀♀, Kuerdening, Gongliu (43.13°N, 82.50°E), 1500 m, 27.VII.1994, coll. Houhun Li and Hongyan Qin; **China, Gansu Province:** 2 ♀♀, Sunan (39.42°N, 98.29°E), 2251 m, 16.VIII.2007, coll. Feng Yang and Hanguang Gao.

#### Diagnosis.

This species is similar to Merulempista cingillella, but differs in the clearly separated discocellular stigmata on the forewing; in the male genitalia the costa with distal process arising from 5/6 of the valva and in the female genitalia the antrum being triangular posterolaterally. In Merulempista cingillella, the discocellular stigma on the forewing is kidney-shaped, the distal process of the costa arises from 3/4 of the valva, and the antrum is elongately leaf-shaped posterolaterally.

#### Description.

Adult (Fig. 4). Wingspan 22.0–27.0 mm. Head greyish brown to dark brown. Antenna with scape greyish brown to dark brown, twice longer than wide; flagellum greyish yellow ringed with brown on dorsal surface, yellowish brown on ventral surface; male sinus with brush of brownish black scales. Labial palpus in male stronger than in female; first segment greyish white, in male mixed with pale ochreous; second and third segments brown, mixed with pale ochreous except greyish white dorsally. Maxillary palpus columniform; in male golden yellow, about equal length to second segment of labial palpus; in female greyish white, slightly longer than third segment of labial palpus. Patagium pale reddish brown. Thorax and tegula brown tinged with greyish white except tegula pale reddish brown at base. Forewing three times longer than wide, apex rounded, termen bluntly oblique; ground coloration greyish brown to brownish black, mixed with reddish brown and greyish white; antemedian line white, extending from 1/4 of costal margin to 1/3 of posterior margin, edged with erect black scales along outside; discocellular stigmata brownish black, clearly separated, forming two distinct spots; postmedian line white, dentate, parallel with termen; termen pale brown to brownish black, discontinuous, sometimes forming small dark spots; cilia grey mottled brown. Hindwing pale grey; cilia yellowish white.

#### Male genitalia

(Fig. 8). Uncus wide at base, narrowed toward bluntly rounded apex, length as long as basal width. Gnathos dilated basally, slender and pointed distally, hooked apically, about 2/5 length of uncus. Costa wider than sacculus; distal process fingerlike, arising from about 4/5 length of valva, forming an acute angle with valva at outside, bearing a needlelike apical prong slightly exceeding end of valva. Valva about four times longer than wide, basal half slightly narrower, widened and arciform ventrally from middle to 5/6; distal 1/6 triangularly shaped, densely covered with long setae, narrowly rounded at apex; clasper finger-shaped, blunt apically; sacculus about half length of valva, basal half slightly wider than distal half. Vinculum broad, U-shaped; anterior margin slightly concave inward at middle. Juxta V-shaped; lateral lobe narrowed distally, curved inward. Phallus about same length as valva, thick at base; with three needle-like cornuti, longest one about 3/5 length of phallus, located medially, other two placed distally. Eighth sternite and culcita shown in figure 8a.

#### Female genitalia

(Fig. 12). Papillae anales triangular, finely haired. Apophyses anteriores shorter than apophyses posteriores, slightly expanded at base. Antrum large, with a longitudinal crevice centrally, triangularly protruding backward posterolaterally. Ductus bursae slightly shorter than corpus bursae; posterior 1/3 membranous, smooth; anterior 2/3 sclerotized, with small pieces of sclerites. Corpus bursae ovate, membranous; large elongate accessory sac from left side of corpus bursae posteriorly, ductus seminalis from its distal part; signum deeply sunken, with numerous coniform spines, placed posteriorly.

#### Distribution.

China (Gansu, Xinjiang).

#### Etymology.

The specific epithet is derived from the Latin *digitatus* (= digitate), in reference to the distal process of costa.

### Merulempista cingillella

(Zeller, 1846)

Figs 5 9 13

Pempelia cingillella Zeller 1846: 779. Syntypes, Type locality: Hungary.
Salebria cingilella : Rebel in Spuler 1910: 211.
Meroptera cingillella : Hannemann 1964: 164.
Merulempista cingillella : Ivinskis 1984: 65.

#### Material examined.

**China, Tianjin:** 5 ♀♀, Dahuangpu (39.26°N, 117.16°E), Wuqing, 15 m, 20–21.VII.2005, coll. Houhun Li; 1 ♀, Beidagang (38.43°N, 117.21°E), 25.VIII.2004, coll. Jiasheng Xu and Jialiang Zhang; 1 ♀, Yadian (38.56°N, 117.14°E), 24.VIII.2004, coll. Jiasheng Xu and Jialiang Zhang; **China, Hebei Province:**1 ♂, 1 ♀, Xiaowutaishan (39.59°N, 114.48°E), Weixian, 1200 m, 24, 28.VII.2000, coll. Yanli Du and Zhendong Li; 1 ♂, Wulingshan (40.38°N, 117.27°E), 900 m, 31.VII.2001, coll. Yanli Du and Zhendong Li; **China, Inner Mongolia Autonomous Region:** 1 ♂, 1 ♀, Chengguanzhen, Dengkou County (40.18°N, 107.00°E), 1000 m, 18.VIII.2002, coll. Zhiqiang Li and Dandan Zhang; 4 ♂♂, 11 ♀♀, Chengguanzhen, Dalateqi (40.24°N, 110.01°E), 960 m, 13.VIII.2002, coll. Zhiqiang Li and Dandan Zhang; 3 ♂♂, 5 ♀♀, Dongfeng (40.53°N, 107.09°E), Hangjihouqi, 1000 m, 20~21.VIII.2002, coll. Zhiqiang Li and Dandan Zhang; 2 ♂♂, 1 ♀, Baliqiao (41.04°N, 108.13°E), Wuyuan County, 960 m, 17.VIII.2002, coll. Zhiqiang Li and Dandan Zhang; 3 ♀♀, Fuxingzhen (40.56°N, 107.56°E), Wuyuan County, 960 m, 16.VIII.2002, coll. Zhiqiang Li and Dandan Zhang; 4 ♂♂, 8 ♀♀, Qianqi (40.75°N, 108.65°E), Bameng, 1075 m, 12.VIII.2006, coll. Zhiwei Zhang; **China, Ningxia Hui Autonomous Region:** 2 ♂♂, Shapotou (37.30°N, 105.11°E), Zhongwei, 1200 m, 10.VIII.2000, coll. Houhun Li and Shuxia Wang; 3 ♀♀, Yuanyichang (37.27°N, 105.42°E), Zhongning, 1170 m, 16–17.VII.1993, coll. Houhun Li; 1 ♂, 1 ♀, Xinpu (37.09°N, 105.43°E), Zhongning County, 1170 m, 26.VII.1993, coll. Houhun Li; 3 ♂♂, 3 ♀♀, Luhuatai (38.37°N, 106.12°E), Yinchuan, 24, 31.VII.1982, coll. unknown; 1 ♂, Yinchuan (38.29°N, 106.13°E), 20.V.1963, coll. unknown; **China, Xinjiang Uygur Autonomous Region:** 4 ♂♂, 2 ♀♀, Jinghe County (44.35°N, 82.53°E), 22.VIII.1994, coll. Duoliken Bashanbayi; 2 ♂♂, 4 ♀♀, Beitun (47.21°N, 87.49°E), 530 m, 20, 22.vii.1994, coll. Houhun Li and Hongyan Qin; 4 ♂♂, 10 ♀♀, Mohe (43.13°N, 82.45°E), Gongliu, 1100–1500 m, 6, 29.VI-7.VII.1994, coll. Xincheng An, Houhun Li and Hongyan Qin; 4 ♂♂, 2 ♀♀, Zepu (38.11°N, 77.16°E) 29.VI-7.VII.1994, coll. Aisihaer Maimaiti; 2 ♂♂, 1 ♀, Ganjiahu (44.54°N, 83.23°E), 27.VI.1984; 1 ♂, 2 ♀♀, Jiashi County (39.29°N, 76.23°E), 1240 m, 17~18.IX.1987, coll. Houhun Li; 1 ♀, Yengisar County (38.55°N, 76.10°E), 1320 m, 15.IX.1987, coll. Houhun Li.

#### Diagnosis.

This species is characterized by the discocellular spots being fused to a kidney-shaped stigma, in the male genitalia by the costa of valva having a distal process straightly bending backward, forming a right angle with the valva, and in the female genitalia by the corpus bursae having a large elongate spined sclerite extending from the posterior end to middle or to near anterior margin.

#### Hostplants.

Myricaria germanica (Linn.), Tamarix gracilis Willd., Tamarix sp.

#### Distribution.

China (Hebei, Inner Mongolia, Qinghai, Ningxia, Tianjin, Xinjiang); Europe: Albania, Austria, Bosnia and Herzegovina, Croatia, France, Germany, Hungary, Italy, Russia, Slovakia, Spain, Switzerland, Turkey, Ukraine (Zeller 1846; Nuss et al. 2010); North Africa: Morocco.

### Merulempista cyclogramma

(Hampson, 1896)

Figs 6 10 14

Phycita (Dioryctria) cyclogramma Hampson 1896: 91. Type locality: Sri Lanka.[Gyrtona cyclogramma: Roesler and Küppers 1979: 78. Misidentification.]
Merulempista cyclogramma : Roesler 1984: 39.

#### Material examined.

**China, Guangxi Zhuang Autonomous Region:** 4 ♂♂, 5 ♀♀, Nanping and Pinglongshan, Shangsi County (22.09°N, 107.59°E), 510–770 m, 3–6.IV.2002, coll. Shulian Hao and Huaijun Xue.

#### Diagnosis.

This species is conspicuously different from its congeners by the costa of valva weakly sclerotized and lacking the distal process, the juxta with gradually broadened, posteriorly rounded and spinulate lateral lobe, and the vinculum with a small papillate anteromedian process in the male genitalia; and by the antrum deeply and widely concave at middle, elongate featherlike posterolaterally in the female genitalia.

#### Distribution.

China (Guangxi); Indonesia (Sumatra), India, Sri Lanka.

#### Remarks.

Of the four species described in this paper, Merulempista cyclogramma is the only one that is distributed in the southern part of China, while the other three occur in the northern and northwestern parts of China (Fig. 2). Taking the genital structures into consideration, the taxonomic position of this species needs further study with additional material.

This species is recorded as new for China.

## Supplementary Material

XML Treatment for Merulempista

XML Treatment for Merulempista rubriptera

XML Treatment for Merulempista digitata

XML Treatment for Merulempista cingillella

XML Treatment for Merulempista cyclogramma
